# Extracellular matrix-modified helix-flexible nerve conduit with optimal mechanics and nerve regenerating properties

**DOI:** 10.1016/j.mtbio.2025.102501

**Published:** 2025-11-07

**Authors:** Yixiao Tan, Jiafeng Yi, Pengtao Shi, Qingda Wei, Yuhui Cui, Yanjun Guan, Haofeng Cheng, Tianqi Su, Qianru Yao, Haolin Liu, Ruichao He, Junli Wang, Yiben Ouyang, Xiaoyang Fu, Jinjuan Zhao, Yu Wang, Qi Quan, Wei Chai

**Affiliations:** a Senior Department of Orthopedics, the Fourth Medical Center of PLA General Hospital, Beijing, 100048, China; b Institute of Orthopedics, Beijing Key Lab of Regenerative Medicine in Orthopedics, Key Laboratory of Musculoskeletal Trauma & War Injuries PLA, No. 51 Fucheng Road, Beijing, 100048, China; c Graduate School of Chinese PLA General Hospital, No. 28 Fuxing Road, Beijing, 100853, China; d School of Medicine, Nankai University, Tianjin, 300071, China

**Keywords:** Umbilical cord mesenchymal stem cells, Extracellular matrix, Helical-structure, Anti-kinking, Peripheral nerve regeneration, Cross-joint nerve injury

## Abstract

Autologous nerve grafting remains the gold standard for peripheral nerve repair, yet its clinical application is limited by donor scarcity and secondary damage. This study aimed to develop a tissue-engineered nerve graft with optimal mechanical properties and bioactivity. By integrating an extracellular matrix derived from human umbilical cord mesenchymal stem cells with helical-structured nerve conduits, we successfully constructed a novel composite conduit. This conduit demonstrated exceptional kink resistance and compressive strength, enabling adaptation to dynamic mechanical environments such as transjoint regions. Furthermore, the ECM modification provided a highly biocompatible microenvironment that significantly promoted Schwann cell proliferation, angiogenesis, and axonal regeneration. In a rat sciatic nerve defect model, the conduit achieved key outcomes—functional recovery, electrophysiological performance, and axonal regeneration density—comparable to those of autografts. This work presents an innovative therapeutic solution with significant clinical translational potential for repairing long-segment and complex nerve defects spanning anatomical joints.

## Highlights

• A helical hUMSC-ECM nerve conduit combines biomimetic structure and bioactive components to enhance regeneration.
• The conduit provides excellent anti-kinking and mechanical strength for dynamic repair across joints.
• The conduit promotes robust axonal regeneration and functional recovery, matching autograft performance.

## Graphical abstract

**Figure:**
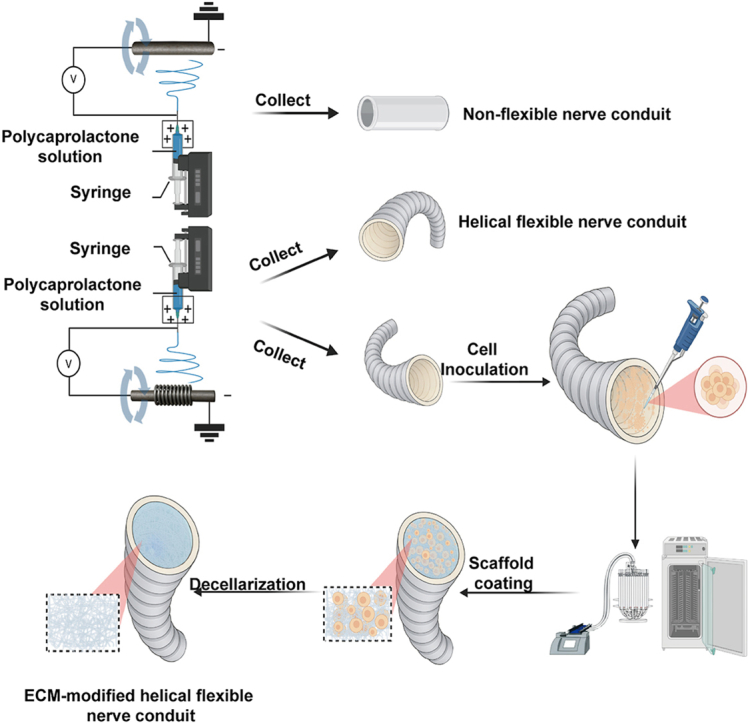

## 1 Introduction

Traumatic Peripheral Nerve Injuries (PNIs) often result in permanent functional impairment and have recently emerged as a major public health concern [1]. Clinically, short-segment nerve defects are usually managed with direct end-to-end neurorrhaphy of transected nerves [2,3]. Conversely, functional recovery remains clinically challenging for extensive nerve gaps or injuries where primary repair is unfeasible—considering the human physiological axonal regeneration rate of ⁓1 mm/day [4,5]. Consequently, Autologous Nerve Grafting remains the primary reconstructive modality for significant PNIs [6,7]. Despite being the gold standard for repairing long-segment defects, Autologous Nerve Grafting is limited in clinical applicability due to donor unavailability and donor-site morbidity [[8], [9], [10], [11]]. Therefore, developing alternative nerve grafts with comparable efficacy to autografts has become a key research priority in neural regeneration medicine.

Advances in tissue engineering and regenerative medicine have yielded novel nerve repair strategies. Tissue-engineered nerve constructs, potential substitutes for Autologous Nerve Grafts, could recapitulate both the physical and biochemical microenvironment of native neural matrices, supporting and guiding axonal regeneration [12]. Conventional tissue-engineered nerve grafts typically comprise biomolecular-based physical scaffolds supplemented with cells/growth-promoting factors [4,13], wherein the Nerve Guidance Conduit (NGC) scaffold serves as the fundamental structural component. These constructs are specifically designed to mimic the three-dimensional (3D) physical microenvironment during nerve regeneration, providing essential structural support and protection for axonal growth. Both natural (e.g., chitosan, silk fibroin) and synthetic (e.g., polycaprolactone, polyglycolic acid) polymers have been extensively explored for neural regeneration applications [[14], [15], [16], [17]], with some clinically validated synthetic polymer-based conduits achieving commercial translation [18]. Due to structural deficiencies, including lumen collapse, reduced effective volume, and suture displacement, conventional NGCs often exhibit limited clinical efficacy [19,20]. When bridging dynamic anatomical regions such as joints, axial tension and torsional deformation during movement often induce lumen collapse, thus impairing nerve regeneration [[21], [22], [23]]. Recent advancements in tissue engineering and regenerative medicine have enabled the precise fabrication of NGCs with optimal mechanical strength and superior biocompatibility, presenting a transformative opportunity for nerve repair. Furthermore, conduit designs with reinforced structures such as corrugations, coils, or helices demonstrated significantly enhanced compressive strength and bending resistance in specialized mechanical environments such as articular regions [24,25]. However, these conventional scaffolds often lack essential bioactive signaling molecules and elicit chronic inflammatory and foreign body responses upon implantation [[26], [27], [28], [29]], limiting their reparative efficacy primarily to small-gap defects. Although their distinct physical architectures could accommodate complex biomechanical environments (e.g., transarticular regions), non-conventional design features may adversely affect endogenous nerve regeneration, highlighting the insufficiency of standalone neural scaffolds to support the regeneration of longer and more complex nerve defects. Researchers have attempted to incorporate bioactive components (e.g., functional cells or neurotrophic factors) into scaffolds to optimize the neural regenerative microenvironment [15,[30], [31], [32]]. However, factors such as low cell viability, poor targeting efficiency of factor delivery, and potential safety concerns could markedly constrain these strategies, limiting their clinical translation [33].

The extracellular matrix (ECM) is a tissue-specific biomacromolecular network composed of secreted proteins and glycosaminoglycans that provides structural support and dynamically orchestrates cellular processes—including proliferation, migration, differentiation, and functional expression—via bioactive signaling molecules [[34], [35], [36], [37], [38]]. As a natural biomaterial, the ECM exhibits inherent biocompatibility, retaining native molecular and architectural features that mimic host tissues and promote bio-communication, thereby mitigating foreign body responses such as fibrous encapsulation [[39], [40], [41]].

Given its multifaceted properties, ECM integration with conduit scaffolds is a viable technique for improving repair outcomes. Previous research predominantly focused on material modification using isolated ECM components (e.g., specific proteins or factors) and examined their individual effects in isolation [42]. This reductionist approach overlooked the synergistic regulatory mechanisms of the ECM as a highly integrated functional system within the microenvironment, failing to fully capture its coordinated regulatory potential. Although chemically decellularized tissue-derived ECM (e.g., acellular nerve matrix) preserves a relatively intact native architecture, donor scarcity, residual immunogenicity risks, and potential pathogen transmission could limit its clinical utility [43]. Cell-derived ECM, particularly stem cell-derived ECM, has recently emerged as a highly promising alternative, primarily due to its controllable production, enhanced biosafety (pathogen-free nature), and dynamic programmability [[44], [45], [46], [47]]. This ECM could be reproducibly prepared under pathogen-free *in vitro* conditions, effectively addressing donor limitations. Moreover, when combined with synthetic materials, it could readily adapt to scaffold architectures while exerting ideal bioactive properties [[48], [49], [50], [51], [52], [53]]. Through specific molecular composition (e.g., characteristic proteoglycans and growth factor networks), it actively modulates cellular behavior and tissue remodeling via biomaterial-cell crosstalk, effectively mimicking the native regenerative microenvironment [[54], [55], [56]]. Therefore, integrating the cell-derived ECM into sophisticated nerve conduit designs could greatly improve repair outcomes for long-segment and anatomically complex nerve defects.

These novel elements and insights should be systematically integrated into the design of current tissue-engineered nerve constructs in order to optimize their performance relative to Autologous Nerve Grafting. Herein, we aimed to develop an advanced composite nerve conduit that integrates exceptional mechanical adaptability with biomimetic bioactivity. Based on the engineering threaded tubing concept, we fabricated a flexible Polycaprolactone (PCL) nerve conduit with helical architecture using electrospinning technology. In our previous study, it was demonstrated that this spiral configuration conferred superior compressive strength and bending resistance, providing mechanical stability for nerve regeneration in dynamic stress environments such as transjoint applications [57,58]. For microenvironment reconstruction, the helical conduits were modified with Human Umbilical Cord Mesenchymal Stem Cell (hUMSC)-derived ECM, creating a highly biomimetic biochemical microenvironment for axon regeneration. According to reports, hUMSCs possess a high proliferative capacity, low immunogenicity, and excellent accessibility, furthermore, their secreted ECM exhibited significant pro-neuroregenerative potential [59,60], making them an ideal ECM source. To evaluate the regenerative efficacy of the hUMSC-ECM-modified Helical Flexible Nerve Conduit (EHNC), we established two parallel control groups: Helical flexible nerve conduits without ECM modification (HNC) and Conventional non-flexible nerve conduits (NNC). The structural attributes of the synthesized materials were characterized using Scanning Electron Microscopy (SEM) and Immunofluorescence (IF) staining. Furthermore, *in vitro* cellular experiments were conducted to explore their effects on functional cells and validate biosafety. A comprehensive multidimensional evaluation was also performed in a rat sciatic nerve defect model (10 mm), incorporating systematic histomorphological, electrophysiological, and functional recovery analyses, to comprehensively examine their nerve regeneration-promoting capacity.

## 2 Results

### 2.1 Material general view and kinking test

**Fig. 1:**
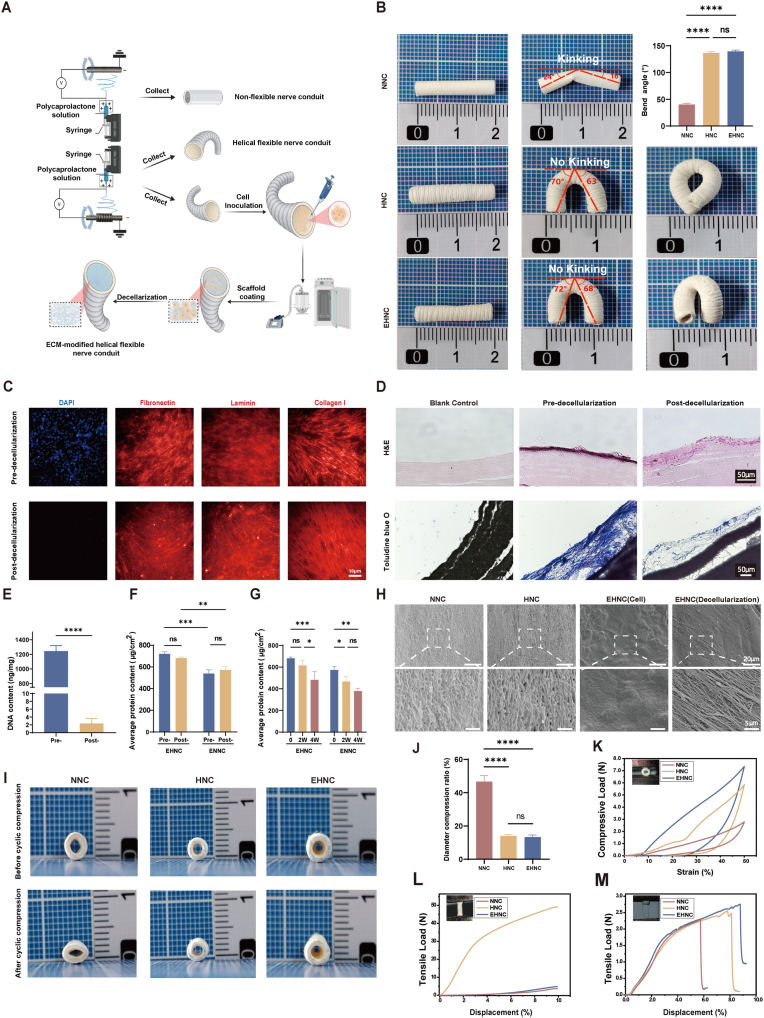
Construction and Characterization of Materials A. Schematic diagram showing the material preparation procedures. B. Macroscopic view and kink resistance of EHNC, HNC, and NNC neural conduits. C. Immunofluorescence (IF) staining images of hUMSC-ECM-modified materials. D. H&E and toluidine blue staining showing the extracellular matrix (ECM) attachment. E. Comparison of DNA content in EHNC materials pre- and post-decellularization. F. Analysis of the changes in protein levels of EHNC and ENNC materials pre- and post-decellularization. G. Comparison of the protein retention rates of EHNC and ENNC materials loaded with ECM at 0, 2, and 4 weeks. H. Scanning electron microscopy (SEM) analysis of surface morphology. I. Changes in the lumen of NNC, HNC, and EHNC materials before and after 1000 compression cycles. J. Compression ratio of lumen diameter for NNC, HNC, and EHNC materials before and after 1000 compression cycles (n = 5). K. Stress-strain curves of NNC, HNC, and EHNC materials during a complete cycle of cyclic compression. L. Stress-strain curves of NNC, HNC, and EHNC materials under 1 cm stretching. M. Analysis of suture retention strength simulating intraoperative suture pull-out. [p < 0.05(∗), p < 0.01(∗∗), p < 0.001(∗∗∗), p < 0.0001(∗∗∗∗)].

Fig. 1B shows the general view of the three types of nerve conduits, with a surface thread structure distinguishing both EHNC and HNC from NNC. To simulate the physiological bending microenvironment during peripheral nerve regeneration, we utilized flexible metal wires as supporting frameworks to progressively apply bending moments until kinking occurred and quantitatively measure the critical kinking angle in order to evaluate the anti-kinking performance of conduits. Both EHNC and unmodified HNC exhibited excellent anti-kinking properties, with 140 ± 3.42° and 133 ± 5.25° critical kinking angles, respectively. Furthermore, both conduits maintained lumen structural integrity even when bent into loops, exhibiting no structural failures such as kinking or collapse. Conversely, conventional NNC displayed significant mechanical deficiencies, developing structural kinking at 40 ± 3.35°, with >50 % reduction in cross-sectional area at the kinking site (p < 0.01). Moreover, upon removal of the bending loads, the NNCs retained substantial plastic deformation attributes, manifesting as irreversible lumen distortion that would severely compromise their clinical applicability.

### 2.2 Characterization of materials

The ECM was systematically subjected to IF staining to detect characteristic ECM structural proteins, including Fibronectin, Laminin, and Collagen type I (Fig. 1C). All three core matrix proteins exhibited typical fibrous network distribution patterns, forming continuous and uniform protein networks on material surfaces. Notably, after decellularization, despite DAPI staining confirming the complete removal of nuclear components, the three characteristic proteins maintained strong specific fluorescence signals. Their spatial distribution patterns and fibrous network topologies also remained intact, with no apparent fragmentation or aggregation observed. These findings conclusively demonstrate that decellularization effectively removed cellular components while effectively preserving both the key protein composition and the ECM's 3D ultrastructure.

Histological analysis further confirmed the ECM modification effect (Fig. 1D). According to the hematoxylin-eosin (H&E) staining results, before decellularization, dense ECM enveloped hUMSCs and covered the inner material surface, distinctly contrasting with the underlying scaffold. After decellularization, hematoxylin-stained nuclei were completely absent while the ECM structure remained intact. To exclude potential interference from scaffold material staining, toluidine blue staining was performed for additional validation. Whereas cell nuclei were clearly visible in the ECM complex before decellularization, they were entirely removed after decellularization. Meanwhile, the ECM's primary structural components were preserved, confirming the thoroughness of decellularization and ECM architecture stability.

Since residual cellular components may elicit host immune rejection and compromise the tissue regeneration microenvironment, we systematically evaluated decellularization efficiency through quantitative assessment of residual DNA content. The decellularized materials exhibited a substantially lower DNA content [2.36 ± 1.10 ng/mg (dry weight)] compared to untreated controls (1243.79 ± 73.82 ng/mg), demonstrating statistically significant differences (p < 0.001) (Fig. 1E). Furthermore, post-treatment DNA content approached the detection limit, indicating the superior thoroughness and consistency of the decellularization protocol.

Quantitative analysis of total protein content in NNCs and HNCs after ECM loading revealed significant differences before and after decellularization. Before decellularization, the EHNC group demonstrated superior protein retention, with a 33.70 % ± 5.28 % higher retention rate compared to the hUMSC -ECM-modified Non-flexible Nerve Conduits (ENNC) group (p < 0.05). Although the post-decellularization protein content changes in both groups did not reach statistical significance (p = 0.4329 and 0.6242 for the ENNC and EHNC groups, respectively), the EHNC group maintained significantly higher protein retention (p < 0.05) (Fig. 1F). Furthermore, even though both materials showed comparable protein retention rates during the 4-week long-term immersion study, the EHNC group ultimately retained more functional proteins due to initial differences in total protein content (p < 0.05) (Fig. 1G). These findings collectively suggest that the helical architecture may enhance ECM-scaffold interactions, thereby more effectively retaining key ECM components. Therefore, nerve conduits with specialized helical structures may serve as superior ECM-modified carriers that can better recapitulate the microenvironmental characteristics of native ECM.

Furthermore, SEM analysis revealed distinct microstructural differences in the inner surfaces of NNC, HNC, and EHNC materials before and after decellularization. Both the unmodified NNC and HNC groups exhibited typical homogeneous electrospun fiber structures, lacking ECM characteristics. Conversely, the EHNC group modified with hUMSC-derived ECM displayed a markedly different morphology—abundant ECM deposits completely enveloped the underlying electrospun fibers. Notably, the EHNC group maintained an intact 3D ECM network structure post-decellularization, with fibrous networks stably attached to the scaffold surface and no residual cellular components observed. These findings demonstrate that hUMSC-derived ECM could effectively reconstruct the scaffold surface microenvironment and retain its structural integrity post-decellularization (Fig. 1H).

### 2.3 Mechanical property testing

We comprehensively evaluated the mechanical properties of three nerve conduits—EHNC, HNC, NNC—through systematic mechanical testing. The lumen compression rates of EHNC and HNC after 1000 compression cycles were maintained at 13.34 ± 1.19 % and 14.03 ± 0.77 % respectively, significantly lower than the NNC group's 46.68 ± 3.23 % (p < 0.001) (Fig. 1I–J). According to the single-cycle compression results, ECM modification further enhanced the radial compression resilience of EHNC compared to the HNC group, with both helical-structured conduits (EHNC and HNC) demonstrating significantly superior performance to conventional NNC conduits (p < 0.01) (Fig. 1K; Video. S1A–C). Besides confirming that ECM modification does not compromise the conduits' radial compression resistance, these findings also suggest that ECM components may reinforce the fibrous network of conduit walls, enhancing compressive performance. In the evaluation of axial tensile mechanical properties, both EHNC and HNC exhibited minimal mechanical response variations at 1 cm axial displacement, whereas NNC required several-fold greater axial force than EHNC and HNC under identical stretching conditions (Fig. 1L; Video. S2A–C). This disparity demonstrates that the helical structural design efficiently distributes axial stress and prevents local stress concentration, thus effectively enhancing the conduits' tensile deformation performance. To better simulate dynamic mechanical environments encountered in clinical settings (particularly periodic stretching across joint regions), we designed suture-fixed tensile experiments. Compared to NNC, both EHNC and HNC demonstrated more uniform stress distribution and superior elastic deformation capacity, sustaining greater tensile deformation before suture rupture (Fig. 1M; Video. S3A–C). These characteristics conferred significant advantages in resisting suture pull-out.

### 2.4 Evaluation of biosafety and effects on neural cell viability

**Fig. 2:**
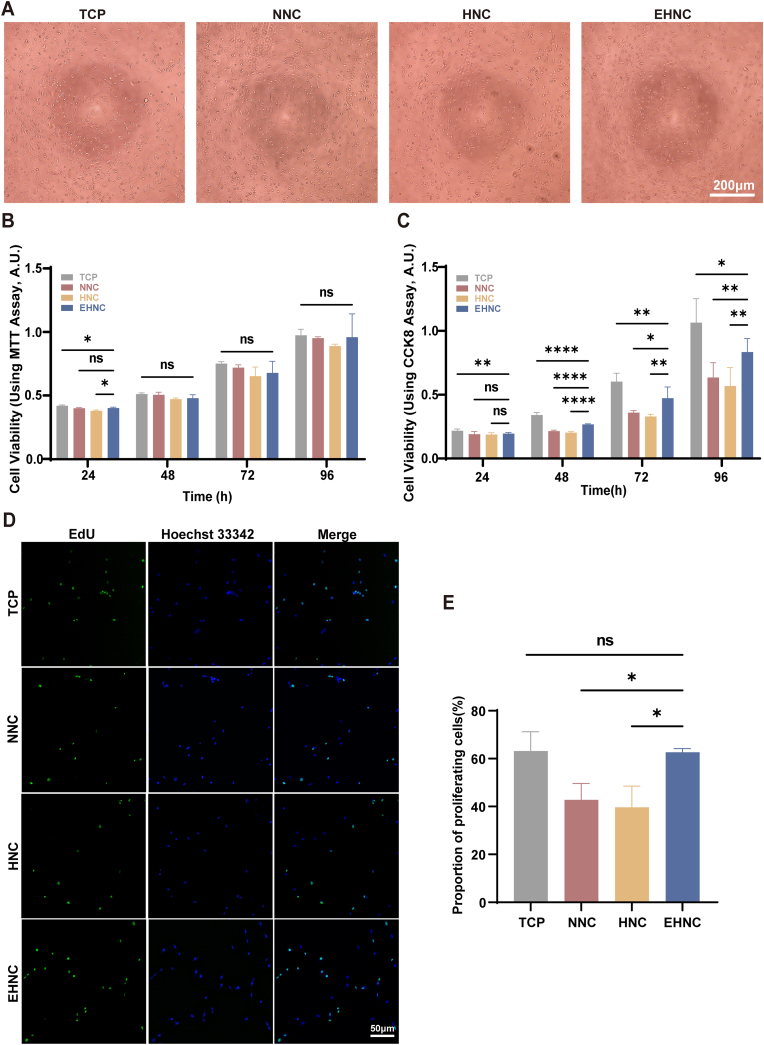
Biocompatibility of Materials and Schwann Cell Growth A. Morphological analysis of L-929 cells cultured with extracts of NNC, HNC, and EHNC materials. B. MTT assay indicating the optical density (OD) of L-929 cells cultured with different material extracts at 24, 48, 72, and 96 h (n = 5). C. Quantitative analysis of Schwann cell proliferation viability on materials (n = 10). D. Immunofluorescence staining images of Schwann cell proliferation in the EdU assay. E. Quantitative analysis of the proliferating Schwann cells (EdU-positive) (n = 3). [p < 0.05(∗), p < 0.01(∗∗), p < 0.001(∗∗∗), p < 0.0001 (∗∗∗∗)].

To systematically evaluate the biosafety of EHNC, we employed standard MTT colorimetric assays for *in vitro* cytotoxicity testing using L-929 cells. Cells cultured with all experimental groups (EHNC, HNC, and NNC) maintained a typical spindle-shaped morphology with well-extended pseudopodia (Fig. 2A), and no significant cell density differences were observed among the groups (p > 0.05) (Fig. 2B). Quantitative analysis of cell viability further demonstrated that the relative cell survival rate was 95.2 ± 2.1 % for EHNC, with both the HNC (91.6 ± 3.4 %) and NNC (95.4 ± 1.8 %) groups also maintaining acceptable viability levels (>90 %), confirming the favorable biosafety profiles of all tested materials, ECM modification did not introduce additional cytotoxic risks. Besides validating the excellent biocompatibility characteristics of EHNC materials, these findings also provide robust experimental evidence supporting their safety for clinical neural repair applications.

Herein, the proliferation kinetics of SCs (Schwann Cells) were monitored through quantitative absorbance measurement at 450 nm (Fig. 2C). We quantitatively analyzed SCs proliferation dynamics at four critical time points (24, 48, 72, and 96 h) during a 96-h culture period. At 24 h, the EHNC, HNC, and NNC groups showed no statistically significant differences in SCs proliferation activity (EHNC vs HNC, p = 0.503; EHNC vs NNC, p = 0.930; HNC vs NNC, p = 0.974). However, after 48 h of culture, the ECM-modified EHNC group began to demonstrate significant pro-proliferative effects (p < 0.05), with a clear hierarchical relationship: TCP > EHNC > NNC > HNC (one-way ANOVA, p < 0.05). All experimental groups exhibited a time-dependent enhancement of proliferation activity with prolonged culture. The pro-proliferative effect of the EHNC group progressively intensified, gradually narrowing the difference with the TCP group, which decreased to 29.1 % ± 15.8 % at 72 h and further to 23.9 % ± 15.6 % at 96 h. These findings strongly indicate that ECM modification reconstructs the cellular microenvironment network, sustainably and significantly promoting SCs proliferation.

To accurately evaluate SC proliferation while excluding non-proliferating cells, we employed EdU staining for direct detection of the proliferative status (Fig. 2D). The EHNC group exhibited an EdU-positive cell rate of 62.6 ± 1.2 %, demonstrating no significant difference from the positive control (TCP groups: 63.17 ± 6.57 %; p > 0.05). Both groups also exhibited significantly higher proliferation rates than the HNC (39.6 ± 7.3 %, p < 0.05) and NNC (42.76 ± 5.61 %, p < 0.05) groups (Fig. 2E). Besides corroborating the MTT assay findings, these results also, through direct DNA replication labeling, confirm the ECM modification-mediated significant proliferative enhancement.

### 2.5 In vivo applicability and repair efficacy evaluation

**Fig. 3:**
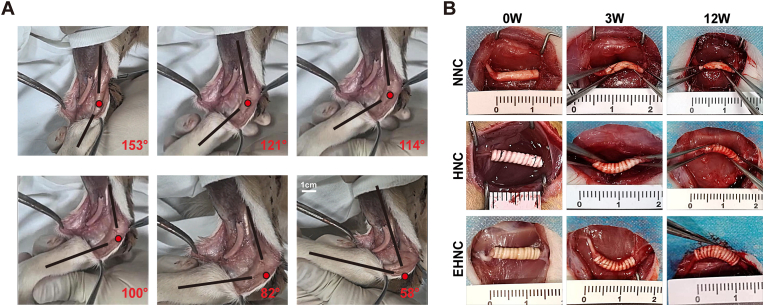
In vivo assessment of kink and compression resistance. A. Schematic illustration showing the EHNC conduit application for beagle tibial nerve defects spanning joint. B. Flexibility testing of NNC, HNC, and EHNC conduits bridging rat sciatic nerve defects at 0-, 3-, and 12-weeks post-operation.

To assess the clinical applicability of EHNC in dynamic physiological conditions, we created a 25 mm tibial nerve defect model spanning the ankle joint in beagle dogs. During maximal ankle joint motion (58° dorsiflexion to 153° plantar flexion), the EHNC conduit exhibited exceptional mechanical adaptability. Neither proximal nor distal ends exhibited significant tension, and no structural failures (including kinking or lumen collapse) were observed throughout the full range of motion (Fig. 3A). These findings indicate that EHNC's unique mechanical properties could enable adaptation to complex biomechanical environments across joint regions, maintaining structural stability while effectively preventing mechanical compression on regenerating nerves, highlighting a significant clinical translation value.

To examine post-implantation mechanical performance changes, three-point bending tests were performed on three types of nerve conduits (EHNC, HNC, NNC) implanted in rat models at 3- and 12-week intervals. It was observed that the EHNC and HNC conduits exhibited excellent anti-kinking properties and structural stability. During uniaxial compression testing, these conduits retained the structures without causing deformation and completely restored their initial morphology after load removal. Moreover, the mechanical properties were stable during the long-term implantation period, without marked degradation observed. In contrast, conventional NNC conduits induced significant structural damages, creating irreversible deformation under lower loads, which were accompanied by permanent lumen collapse (Fig. 3B). Collectively, these findings demonstrate that the helical design provided several mechanical advantages, including achieving superior deformation recovery capacity, long-term implantation stability, and dynamic environmental adaptability, all of which ensured high clinical efficacy of the transjoint nerve repair applications.

**Fig. 4:**
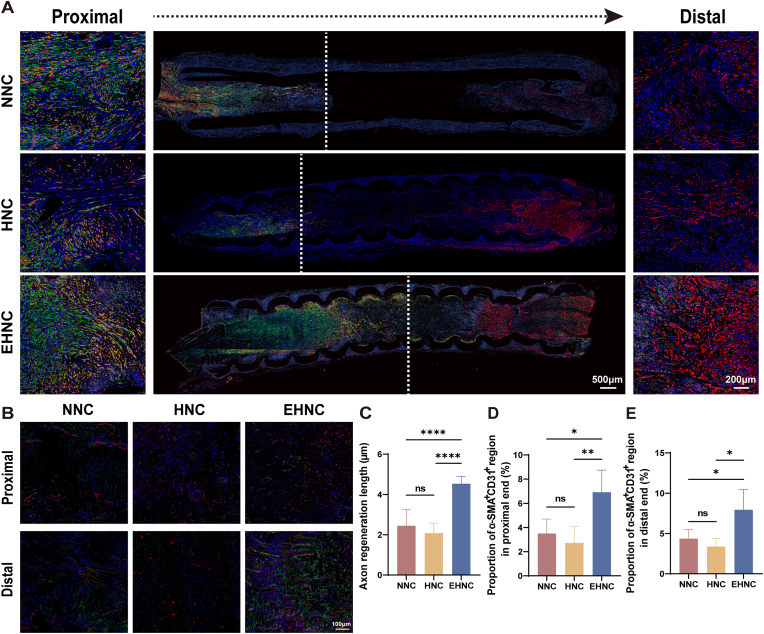
Analysis of nerve regeneration at 3 weeks post-implantation. A. Longitudinal immunofluorescence (IF) staining images of nerve grafts at 3 weeks (Schwann cells: S100-β, red; axons: NF200, green; nuclei: DAPI, blue). White dashed lines indicate axonal regrowth length; insets show magnified proximal/distal regions. B. Representative IF images of vascularization in graft proximal/distal regions (endothelial cells: CD31, green; α-smooth muscle actin: α-SMA, red; nuclei: DAPI, blue). C. Quantification of axonal regrowth length (n = 6). D-E. Percentage area of CD31+/α-SMA + regions in proximal/distal longitudinal sections. [p < 0.05(∗), p < 0.01(∗∗), p < 0.001(∗∗∗), p < 0.0001 (∗∗∗∗)].

Histopathological Evaluation:At 3 weeks post-implantation (early regeneration phase), nerve conduit segments were collected for subsequent longitudinal immunofluorescence staining (S100-β/NF200, CD31/α-SMA) to examine the axonal extension length (reflecting early regeneration rate) and vascular infiltration (Fig. 4A–E). Results of the axonal regeneration analysis demonstrated that the EHNC group promoted axonal growth, with a length of 4.53 ± 0.36 mm, which exceeded the results obtained in the HNC (2.08 ± 0.5 mm) and NNC groups (2.45 ± 0.74 mm). However, there was no significant difference between the latter two groups (p = 0.5347) (Fig. 4A,C). Furthermore, vascular density analysis revealed that the EHNC group had a denser vascular network at both proximal and distal conduit regions (p < 0.05), but that for the HNC and NNC groups was lower, and the difference was not significant (p > 0.05) (Fig. 4B,D-E). These findings indicated that the ECM may improve the nerve repair by optimizing the microenvironment and promoted vascular regeneration.

**Fig. 5:**
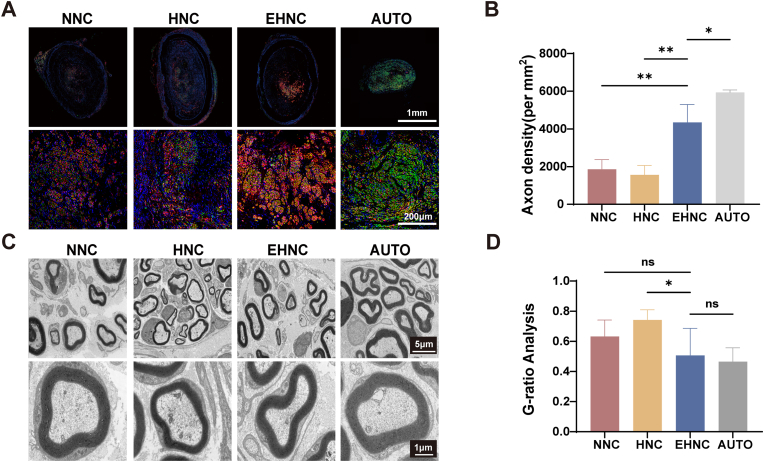
In Vivo Evaluation of Sciatic Nerve Defect Repair Efficacy. A. Mid-graft cross-sectional IF staining at 12 weeks (Schwann cells: S100-β, red; axons: NF200, green; nuclei: DAPI, blue). B. Statistical analysis of axons density in mid-graft cross-sections (n = 3). C. Representative TEM images of the regenerated axons and myelin sheaths. D. G-ratio quantification based on TEM (n = 5). [p < 0.05(∗), p < 0.01(∗∗), p < 0.001(∗∗∗), p < 0.0001 (∗∗∗∗)].

The mid-portion of nerve conduits was investigated during the 12-week post-implantation given that the axonal density per unit area in this region provides the most objective indication of the long-term peripheral nerve regeneration potential (Fig. 5A). Quantitative immunofluorescence analysis revealed that the autologous nerve graft (AUTO, the gold standard for nerve repair) had optimal regenerative outcomes in the mid-portion, with an axonal density of 5931.7 ± 110.8 fibers/mm^2^. Among the engineered nerve conduits, the EHNC group displayed regeneration performance closest to that of AUTO group, achieving an axonal density of 4347.88 ± 778.3 fibers/mm^2^, which was significantly superior to HNC (1560.77 ± 408.25 fibers/mm^2^) and NNC (1854.32 ± 421.93 fibers/mm^2^) groups (p < 0.01) (Fig. 5B). Notably, the EHNC group not only demonstrated excellent axonal regeneration but also showed significant spatial colocalization between Schwann cells and regenerating axons(Fig. 5A), further confirming the unique advantages of this conduit material in facilitating the establishment of a pro-regenerative microenvironment.

Histomorphometrical evaluation of the regenerated nerve. To examine the distal nerve regeneration potential, we examined the ultrastructure of regenerated nerve myelin sheaths through lead-uranium staining combined with transmission electron microscopy(Fig. 5C). Quantitative analysis of the G-ratio (the ratio of axon diameter to total fiber diameter), a key indicator of myelination degree, demonstrated that the EHNC group (0.5068 ± 0.1681) did not significantly differ compared to either the NNC group (0.6324 ± 0.1089) or the gold-standard AUTO group (0.4652 ± 0.0865) (p > 0.05). Notably, the EHNC group had better myelination capability relative to the HNC group (0.7428 ± 0.0659) (p < 0.05), which showed that ECM modification promoted myelin repair and regeneration(Fig. 5D).

**Fig. 6:**
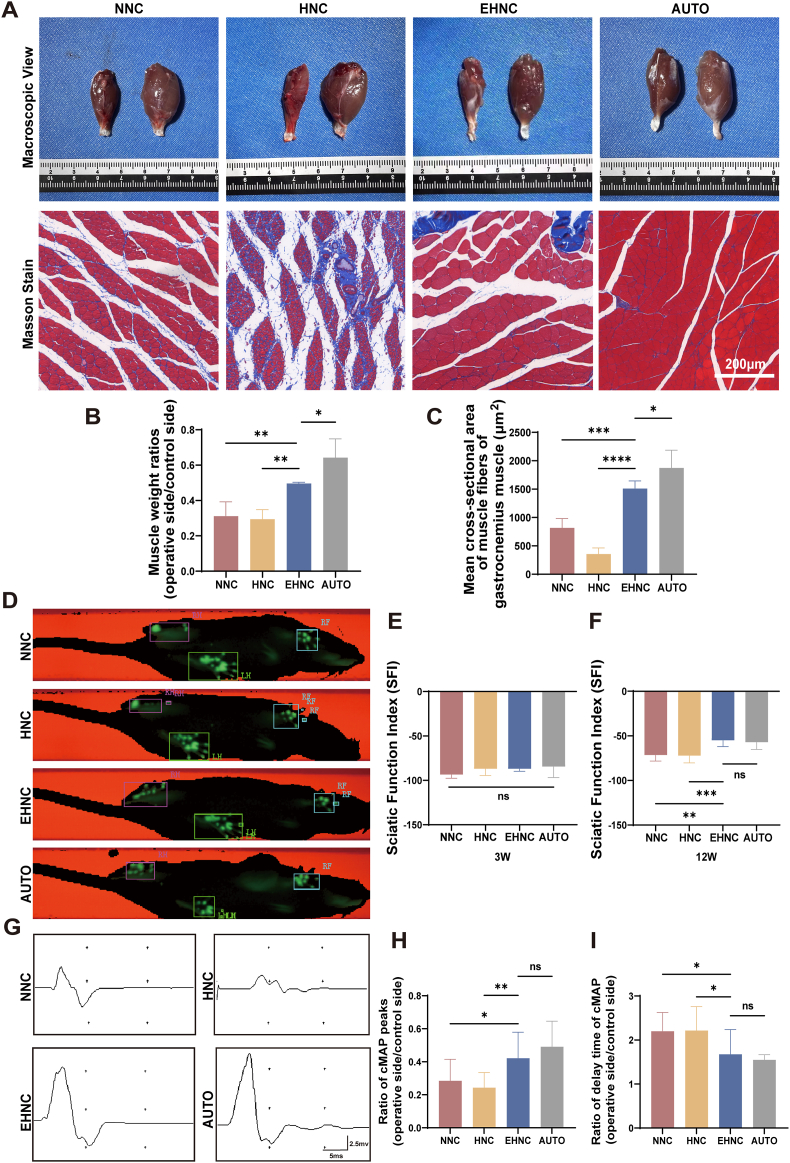
Behavioral and Functional Evaluation Following Sciatic Nerve Repair. A. Gross examination and the Masson staining (blue: collagen fibers; red: muscle fibers) images of the operated-side gastrocnemius muscles in SD rats from the indicated groups at 12 weeks post-operation. B. The recovery rate of wet weight (operated side/control side) of gastrocnemius muscles in each group (n = 5). C. Mean cross-sectional area (CSA) of muscle fibers in the mid-belly region of gastrocnemius muscles for each group (n = 5). D. Two-dimensional footprint photographs of rats at 12 weeks post-operation. LH: left hindlimb (healthy side control); RH: right hindlimb (operated side). E. Sciatic functional index (SFI) of rats in each group at 3 weeks post-repair (n = 5). F. Sciatic functional index (SFI) of rats in each group at 12 weeks post-repair (n = 5). G. Representative cMAP waveforms for each group. H. Statistical analysis of cMAP peaks ratio (operated side/control side) for each group (n = 4). I. Statistical analysis of cMAP delay time ratio (operated side/control side) for each group (n = 4). [p < 0.05(∗), p < 0.01(∗∗), p < 0.001(∗∗∗), p < 0.0001 (∗∗∗∗)].

Target Organ Recovery Evaluation:Sciatic nerve regeneration can delay target muscle denervation atrophy. Therefore, the effect of sciatic nerve regeneration was investigated by conducting quantitative analysis of muscle wet weight ratio and Masson staining. It was observed that the AUTO group (gold standard) had superior muscle wet weight ratio (0.6427 ± 0.097) relative to other groups. Although the muscle wet weight ratio value in the EHNC group (0.4958 ± 0.0067) was lower compared to that in the AUTO group (p = 0.02), it outperformed the HNC (0.296 ± 0.049) and NNC groups (0.311 ± 0.073) (p < 0.01) (Fig. 6A-B). Morphometric analysis further indicated that the AUTO group achieved optimal single-view muscle fiber area (1872.34 ± 283.95 μm^2^), followed by EHNC (1509.44 ± 125.23 μm^2^), which represented a 320 % improvement over the HNC group (355.85 ± 104.45 μm²) and an 85 % improvement over the NNC group (815.28 ± 165.31 μm²), respectively. (Fig. 6C).

Functional Recovery Assessment:At 12 weeks after the 10 mm sciatic nerve defect repair, two-dimensional footprint analysis showed that the operated-side footprints of rats in the EHNC group were clearer and exhibited more pronounced toe stress compared to those in the NNC and HNC groups. In contrast, the footprints of rats in the NNC and HNC groups displayed stress concentrated mainly in the plantar region.(Fig. 6D). Results of the quantitative functional recovery analyses based on the Sciatic Function Index (SFI) indicated that there were no significant differences among the groups (including AUTO, EHNC, HNC, and NNC) at 3 weeks post-operation (p > 0.05), suggesting comparable early-stage functional recovery (Fig. 6E). At the 12 weeks’ time-point, the EHNC group (−55.0357 ± 7.3176) revealed equivalent SFI values to the gold-standard AUTO (−57.1967 ± 7.8619) (p = 0.996), and showed significantly superior recovery compared to the HNC (−72.2150 ± 8.3003) (p < 0.001) and NNC (−71.5843 ± 6.5583) (p < 0.01) groups, translating to nearly 23 % functional improvement over the HNC and NNC groups (Fig. 6F). These results indicate that the ECM-modified EHNC conduits achieved near-autograft-level efficacy in motor function restoration.

Electrophysiological Functional Assessment:Compound muscle action potential (cMAP) analysis of the target gastrocnemius muscle objectively indicated the functional recovery status post-nerve regeneration. Successful regeneration is characterized by complete restoration of two key electrophysiological parameters: a shortened latency period and an increased amplitude, both approaching levels observed in healthy controls. At 12 weeks post-repair, analysis of the cMAP waveforms in the EHNC group closely resembled those seen in the AUTO group (Fig. 6G). Quantitative analysis demonstrated the following: 1) The NNC and HNC groups had lower peak action potentials compared to the AUTO group, but it was not significantly different between the EHNC group and the AUTO group (p = 0.5186) and was significantly better than that of the NNC (p < 0.05) and HNC groups (p < 0.01) (Fig. 6H); 2) Regarding the delay time ratios, EHNC groups achieved equivalent recovery to AUTO groups (p = 0.8769) (Fig. 6I). Analysis of the comprehensive electrophysiological data suggested that the ECM-modified EHNC conduits yielded comparable functional recovery to the AUTO group, exhibiting better parameter restoration than unmodified HNC and NNC groups. Altogether, these findings confirmed that the ECM modification enhanced the conduit repair efficacy, generating functional outcomes similar to the clinical gold standard.

## 3 Discussion

Peripheral nerve injuries are a major global public health challenge as they are associated with high disability rates, substantial treatment costs, and significant socioeconomic burden [61]. Although autologous nerve transplantation is still the mainstay treatment for nerve defect repair [62], its clinical use is limited by several factors, such as donor site morbidity and limited availability. To address these concerns, researchers are increasingly searching for effective alternative solutions. The rapid development of tissue-engineered nerve grafts (TENGs) has shown great potential to overcome these constraints. From early hollow conduits to contemporary composite grafts incorporating cellular and growth factor components [4,13], the philosophy guiding their design focuses on two core elements: mechanical support structures and bioactive microenvironments. However, the available technologies are limited by challenges such as conduits with solely mechanical support functions having limited performance in long-gap regeneration, and over-emphasizing their bioactive optimization may compromise mechanical performance required for dynamic injury environments (e.g., transjoint regions). To address these concerns, it is imperative to develop nerve grafts with optimal mechanical properties and bioactivity, which is a major challenge in the field. Our study directly addresses this dilemma by synergistically integrating a biomimetic helical topological structure with bioactive human umbilical cord mesenchymal stem cell (hUMSC)-derived extracellular matrix (ECM), thereby achieving simultaneous enhancement of mechanical adaptability and regenerative bioactivity.

The main innovation of this work lies not only in the combination of the two functional components, but also in their functional complementarity and mutual enhancement, creating a regenerative microenvironment that surpasses a simple additive effect. The commonly used nerve conduit designs aim to improve the regenerative microenvironment by optimizing the bioactive components and leveraging on oversimplified stiffness augmentation strategies to maintain hollow cylindrical structures. However, this design paradigm does not account for the dynamic requirements of nerve regeneration: although prolonged immobilization post-implantation may temporarily preserve conduit integrity, it causes tissue adhesion and functional impairment [63]. During postoperative recovery, implanted conduits are exposed to compressive, bending, and erosive forces generated by the surrounding tissues and physiological environments [[64], [65], [66]], which can causes irreversible structural damage to conventional designs. In specialized anatomical locations such as transarticular regions, traditional conduits do not efficiently adapt to movement-induced cyclic tensile stresses, which is a major structural limitation to their clinical adoption. Therefore, there is higher reliance on autografts despite their well-documented constraints [21].

Previous studies, particularly the pioneering work of Quan et al. [58], have confirmed that helical groove structures can significantly enhance the compressive strength and bending resistance of nerve conduits, providing an important mechanical solution for the field. However, such research primarily focused on optimizing the material's mechanical properties through topology, without addressing the bioactive requirements of the neural regeneration microenvironment. Building upon this mechanical innovation, our study achieves a critical breakthrough: the functional integration of bioactive hUMSC-derived ECM with the helical flexible nerve conduit. We not only retained the excellent mechanical properties endowed by the topological structure but, more importantly, successfully constructed a composite repair system that combines mechanical adaptability and biocompatibility. This system can not only maintain a stable structural channel but also provide active microenvironmental support for the regeneration process, thereby offering a potential solution to complex nerve repair challenges.

The helical flexible nerve conduit (HNC) provides a mechanically robust and kink-resistant scaffold, as demonstrated by our kinking tests and mechanical evaluations (Fig. 1B,I-M), maintaining excellent structural integrity under compression, tension, and bending. This effectively addresses the issues of lumen collapse and irreversible deformation observed in traditional non-flexible nerve conduits (NNC), representing a key advantage for applications spanning mobile joints, which was further validated in a beagle tibial nerve model (Fig. 3A).

Although the helical topological design can improve the clinical applicability of nerve conduits, this unique architecture can potentially alter the axonal extension processes. Comparative analysis using conventional nerve conduit scaffolds revealed that helical conduits may have a higher actual path length at equivalent macroscopic dimensions owing to their unique groove configurations. Although the groove dimensions are negligible at the macroscopic scale, they create path elongation effects at cellular migration scales. Therefore, although preserving the excellent mechanical properties of helical structures is important, bioactive modification strategies that optimize the regenerative microenvironment are needed to achieve a coordinated improvement of the mechanical support performance and neural regeneration efficiency.

Traditional cell transplantation and growth factor delivery strategies face significant clinical translation challenges due to their inherent limitations such as high preparation costs and unstable bioactivity [33]. In contrast, ECM, as the core component of native cellular microenvironments, can regulate cellular behavior and tissue regeneration processes. Research has confirmed that human umbilical cord mesenchymal stem cells and the bioactive substances they produce possess excellent pro-regenerative efficacy [67], and human umbilical cord mesenchymal stem cell (hUMSC)-derived ECM can enhance neural regeneration [59,60]. Therefore, we utilized hUMSCs with high bioactivity, reliable sourcing, and standardized preparation potential as the optimal donor cell sources for ECM. Thus, modifying the material with hUMSC-derived ECM is key to achieving the synergistic improvement of mechanical adaptability and regenerative bioactivity.

Building upon the HNC, the hUMSC-derived ECM coating transforms this non-bioactive, inert scaffold material into a highly bioactive interface. It mimics the native neural microenvironment, as confirmed by the abundant presence of key structural proteins (such as fibronectin, laminin, and collagen type I) even after decellularization (Fig. 1C), and directly promotes Schwann cell proliferation *in vitro* (Fig. 2C–E). These findings align with the previously reported mechanisms of ECM-mediated enhancement of tissue regeneration by Xiao et al. and Guan et al [60,68]. This indicates that this stable functional modification strategy not only effectively addresses the potential inhibitory effect of the helical structure on regeneration but, more importantly, constructs a highly biomimetic ECM microenvironment, providing an ideal biological foundation for nerve regeneration.

Crucially, we found that these components do not act independently; they exhibit a structure-bioactivity coupling effect. The helical topology with micron-scale grooves significantly increased the loading efficiency and retention rate of ECM proteins. This suggests that the physical design actively contributes to stabilizing the biochemical microenvironment, creating a more durable reservoir of bioactive signals. This finding reveals the structural advantage of the helical configuration in ECM loading: it serves not only a mechanical purpose but also acts as an optimized platform for bioactive functionalization. Furthermore, contrary to concerns that ECM modification might weaken mechanical properties, our results indicate that the ECM reinforced the conduit wall, showing improved radial compressive strength compared to unmodified HNC (Fig. 1K). This mechanical enhancement effect may stem from the ECM-mediated filling of the electrospun fiber micropores combined with the structural reinforcement from the fibrous wall network, suggesting potential synergistic interactions at the microstructural level.

Therefore, the ECM-modified helical flexible nerve conduit (EHNC) represents a highly integrated biomimetic system. Experimental data from a rat model with a 10-mm sciatic nerve defect fully validated its repair efficacy: by 3 weeks post-operation, the EHNC group already showed significant leads in axonal regeneration efficiency and multi-regional vascular density; systematic analysis continued until 12 weeks further demonstrated that this group achieved key metrics—such as electrophysiological signal conduction, functional recovery indices, and regenerated axon density—comparable to those of AUTO groups (Fig. 4A–E, Fig. 5A–D, Fig. 6A–I). This comprehensive repair outcome is rooted in EHNC's unique "macro-micro" dual-design concept: at the macroscopic level, its helical topology effectively disperses mechanical stress and resists external compression, constructing a stable and protected physical channel for nerve regeneration; at the microscopic level, the hUMSC-derived ECM precisely constructs a functionalized regenerative microenvironment, coordinating Schwann cell activation, axonal extension, and the construction of nascent vascular networks through the sustained provision of bioactive signals. This strategy of integrating physical structure with biological matrix successfully transcends the core limitation of segregated mechanical performance and biological function in traditional systems. Ultimately, it is the highly unified synergistic effect between macroscopic protection and microscopic promotion that underpins the key foundation for EHNC's achievement of neural functional reconstruction.

From a fundamental research perspective, an ideal tissue engineering scaffold may perfectly mimic the natural tissue regeneration microenvironment in terms of physical support and biocompatibility. Although the designed ECM-modified helical flexible nerve conduit achieved excellent macroscopic mechanical properties (compression and bending resistance), its cell-scale mechanical microenvironment was not adequately characterized. The study by Guilak et al. showed that the ECM stiffness is a major determinant of stem cell fate [69]. Consistent with the reports of Xu et al., hUMSCs biological behavior is regulated by substrate stiffness [70], which indicates that our ECM may have undergone property alterations due to variations in the base material stiffness. Furthermore, Harris et al. revealed that matrix stiffness directly influenced the Schwann cell regenerative efficiency [71], which implies that hUMSC-derived ECM may influence neural regeneration outcomes via the mechanotransduction mechanisms. However, in this study, precise characterization of the cell-scale mechanical microenvironment was challenging owing to the material's unique topological structure and multiparameter coupling effects. From a long-term perspective of clinical translation, an ideal nerve conduit's lifecycle should match the nerve regeneration process: it should provide a stable physical channel and mechanical support during the early stages of regeneration, then degrade in a timely manner after the nerve has restored its function, ceding space to the new tissue and avoiding long-term foreign body reactions and potential nerve compression risks [66]. The polycaprolactone (10.13039/100018919PCL) material used in this study, with its inherent slow degradation profile (requiring 2–3 years for complete degradation) [72], constitutes one of the main potential limitations for its clinical translation. Although its stable mechanical performance over the 12-week observation period in rats is a notable advantage, considering the longer (typically year-scale) complete regeneration cycle in humans, the long-term retention of PCL scaffolds could introduce a series of uncertainties: First, it might induce a persistent, low-grade chronic foreign body reaction, disrupting the stability of the regenerative microenvironment. Second, after complete nerve regeneration, the non-absorbed scaffold may lose its supportive role and instead become a physical barrier, limiting the further maturation and remodeling of the regenerated nerve. Finally, in young patients or dynamic anatomical locations, non-degradable residues could increase the risk of long-term complications, such as fibrous encapsulation or adhesion. Therefore, although the long-term stability of PCL proved beneficial for bridging the critical regeneration window in this study, developing conduits with tunable degradation rates will be a key frontier direction for advancing this technology towards broader clinical applications. This study validated the efficacy in rodent models and the applicability in a beagle model, but further systematic research is necessary to achieve clinical translation. Future work needs to focus on conducting long-term (≥12 months) safety and efficacy evaluations in large animal models (such as beagles, pigs, or non-human primates), optimizing scaled-up production processes compliant with composite medical device standards to ensure batch consistency, and exploring its applicability in more types of nerve injuries, with the ultimate goal of advancing EHNC from experimental research to clinical application, providing patients with a reliable alternative for nerve repair.

## 4 Conclusion

This study successfully developed and validated an ECM-modified helical flexible nerve conduit. The results demonstrate that this composite conduit effectively resolves the longstanding dilemma of balancing mechanical performance and bioactivity in traditional nerve guides through its unique "macro-micro" synergistic design: the macroscopic helical structure provides mechanical stability and anti-collapse capability in dynamic environments, while the microscopic ECM coating constructs a biomimetic regenerative microenvironment that actively guides cellular behavior and tissue regeneration. Ultimately, it achieved repair efficacy comparable to autografts in a rat model. The broader significance of this work lies in demonstrating a "structure-function" integrated design paradigm for tissue-engineered scaffolds, offering insights for repairing other load-bearing or dynamic tissue defects. However, limitations regarding the complex topology, PCL's long-term degradation, and absent long-term large-animal data necessitate future work to elucidate mechanobiological mechanisms, develop tunable materials, and establish conclusive safety and efficacy profiles in large models.These efforts will advance this promising technology toward clinical application, offering new hope for patients with peripheral nerve injuries.

## 5 Materials and methods

### 5.1 Fabrication of Helix-flexible nerve conduits (HNCs)

To fabricate HNCs, PCL (440744, Sigma-Aldrich) was first dissolved in Hexafluoroisopropanol (HFIP, Aladdin) to prepare an 8 % (w/v) polymer solution. Subsequently, to ensure complete homogenization, the solution was magnetically stirred at 25 °C for 8 h. The electrospinning process was performed using an ET-2531 electrospinning system (Yongkang, China). A 2.5-mL syringe fitted with a 0.4-mm stainless steel needle was then loaded with the PCL solution and mounted on a syringe pump. Electrospinning was conducted at a flow rate of 1.5 mL/h, a working distance of 15 cm, and with the collector rotating at 70 rpm. Furthermore, the electrical configuration comprised +15.5 kV and −7.5 kV applied to the needle tip and custom-designed spiral-patterned collector, respectively. The ambient temperature for electrospinning is set to 25 °C, with a humidity of 30 %. This setup yielded flexible nerve conduits (1.2 cm in length, with 2 mm and 2.5 mm inner and outer diameters, respectively) with distinct spiral microgrooves along the luminal surface. For control experiments, NNCs were fabricated under identical processing conditions, but with a smooth-surfaced collector rather than a spiral-grooved collector. Fig. 1A illustrates the manufacturing process.

### 5.2 Construction of hUMSC-ECM modified HNCs

After vacuum-drying for 24–48 h to remove residual solvents, the prepared HNCs were immersed in 75 % ethanol for 2 h to eliminate bacterial and endotoxin contamination. Subsequently,the samples were subjected to five thorough rinses in sterile PBS. Each rinse was conducted for a duration of 10 min. Following that, the HNCs were placed in low-adhesion 6-well plates and sterilized via irradiation. The processed HNCs were then coated with a 0.2 % gelatin (G7041, Sigma) solution at 37 °C for 4 h before washing three times with PBS to remove excess gelatin solution. Meanwhile, hUMSCs at passage 2 (P2) and 80–90 % confluence were enzymatically dissociated using 0.25 % trypsin-EDTA and resuspended at a density of 5 × 10^5^ cells/mL in complete hUMSCs culture medium (RP 02010, Nuwacell). Subsequently, the cell suspension was carefully seeded onto the HNC housed in low-adhesion 6-well plates. To sustain continuous proliferation, the cells were cultured at 37 °C in a 5 % CO_2_ atmosphere, with the medium being replaced every 48 h. After 12 h of initial incubation to allow cell attachment, the 6-well plates with HNC were rotated 180°to facilitate uniform cell distribution. Another 100 μL of fresh cell suspension (5 × 10^5^ cells/mL) was then added onto each HNC system and cultured for an additional 12 h under standard conditions. The cellular constructs were then transferred aseptically to a perfusion-based rotary bioreactor (Synthecon Inc., Friendswood, TX) for dynamic culture under continuous nutrient perfusion. During the 14-day culture period, the constructs’ luminal surfaces were gently perfused daily using a 2.5 mL syringe to prevent cellular aggregation.The complete medium for hUMSCs cultures was replaced every 48 h. The fresh medium was supplemented with 50 μM L-ascorbic acid (Sigma-Aldrich) to provide optimal growth conditions and encourage ECM production. To ensure complete decellularization of the materials, after culture completion, all culture media were first removed, and the materials were gently rinsed with sterile PBS on both the inner and outer surfaces. Subsequently,to ensure complete decellularization, the materials were first treated with a buffer (0.5 % Triton X-100 and 20 mM ammonium hydroxide in PBS) for 10 min at 37 °C, followed by incubation with 150 U/mL DNase I (D5025, Sigma) for 3 h at the same temperature. This sequential process effectively removed cellular debris and residual nucleic acids. After processing, the samples were gently rinsed three times with PBS to obtain decellularized EHNCs. Fig. 1A details the relevant preparation process. The final products were stored in sterile physiological saline at 4 °C, awaiting subsequent experimental use.

### 5.3 Material general view and kinking resistance test

Herein, three distinct nerve conduit types were fabricated for comparative analysis: EHNCs, HNCs, and NNCs. The gross morphological features of each conduit type were documented through initial macroscopic evaluation using a digital camera (FUJIFILM X100, Japan). A flexible guidewire was inserted into each of the three neural conduits and progressively bent until kinking, with the bending angles for both the guidewire and corresponding conduits recorded.

### 5.4 Immunofluorescence (IF) staining of the ECM

Following fixation with 4 % paraformaldehyde (PFA), both decellularized and non-decellularized EHNCs were blocked for 30 min using diluted goat serum (1:20; Solarbio, SL038). The samples were then incubated for 12 h at 4 °C with primary rabbit polyclonal antibodies targeting Collagen Type I (Col I; Abcam ab270993), Fibronectin (FN; Abcam ab2413), and Laminin (LN; Abcam ab11575). Subsequently, a 2-h incubation with Alexa Fluor 594-conjugated goat anti-rabbit IgG secondary antibody (1:200; Abcam ab150080) was performed at room temperature (RT, 20–25 °C). Finally, nuclei were stained with DAPI (4A Biotech, 139-100), and images were acquired using a 3DHISTECH Pannoramic panoramic confocal scanner.

### 5.5 Histological staining (H&E and Toluidine Blue staining)

For H&E staining, the decellularized and non-decellularized EHNC and HNC without ECM modification were rinsed with PBS on both the inner and outer surfaces and fixed in a 4 % PFA solution (10 times the sample volume) at 4 °C for 24 h. The fixed samples were then dehydrated in 30 % sucrose solution until complete sedimentation. Following embedding in compound gel, the conduits were snap-frozen in liquid nitrogen and serially sectioned into 10-μm cross-sections. These sections were then floated in distilled water for 2 min prior to being stained with hematoxylin for a duration of 2–20 min. After washing off the excess stain, a differentiation solution was applied for 30 s. The sections were then washed with tap water, stained in eosin solution for 60 s, rinsed, and rapidly dehydrated using a graded ethanol gradient (80 %, 90 %, and 100 %). After clearing in xylene for 5 min, the sections were mounted with neutral balsam and examined under a microscope.

The protocol for Toluidine blue staining consisted of a 2-min immersion in distilled water followed by application of a 0.1 % toluidine blue solution for 5 min at room temperature. The sections were then differentiated in 1 % glacial acetic acid for 5–10 s and immediately rinsed gently with distilled water 3–5 times to completely remove residual acetic acid. The rinsed sections were then processed through a graded ethanol series (80 %, 90 %, 100 %) for dehydration, xylene for clearing (5 min), and were finally mounted in neutral balsam for microscopic examination.

### 5.6 Quantification of residual DNA in decellularized materials

Both decellularized and non-decellularized EHNCs (n = 5/group) were gently rinsed three times with deionized water, lyophilized for 24 h in a freeze dryer, and fragmented for weight measurement. After total DNA extraction using the QIAcube HT nucleic acid extraction system (QIAGEN), DNA concentration was determined with a fluorometric quantification kit (P7589, Invitrogen) by measuring fluorescence signals (excitation/emission: 480 nm/520 nm) on an Infinite 200 Pro microplate reader (TECAN) and calculating values based on a standard curve.

### 5.7 Protein retention capacity and duration of ECM-modified materials

The ECM-modified materials' protein retention capacity and duration were evaluated with the Bicinchoninic Acid (BCA) assay [73], using HNC and NNC materials of identical length and inner and outer diameters. After calculating their respective total surface areas, EHNCs and ENNCs were prepared using the same concentration of hUMSCs with the same ECM modification duration. The ECM-modified materials were then placed in 6-well plates containing 2 mL of sterile PBS and shaken horizontally at 100 rpm for 4 weeks at 37 °C, with daily PBS replacement. Three parallel samples were prepared for each group, and protein extraction was performed using the protein extraction kit (WB3050, NCM Bio). For both the decellularized and non-decellularized materials and the samples immersed for 2 and 4 weeks, the materials were gently washed three times with ice-cold PBS before mincing and placing the samples in centrifugal filter columns. An equal volume of denaturing tissue and cell lysis buffer was then added, followed by 20 homogenization cycles using a grinding pestle. After centrifugation of the lysates (12,000–14,000×*g*, 1 min), the supernatant was collected for total protein quantification using a BCA assay kit (NCM Bio, WB6501) per the manufacturer's protocol. The protein content per unit area was determined by measuring the absorbance at 562 nm on a microplate reader and comparing the values to a standard curve.

### 5.8 Scanning Electron Microscopy (SEM) detection

For ultrastructural comparison, four experimental groups were processed concurrently: (1) Pre-decellularization EHNC, (2) post-decellularization EHNC, (3) HNC, and (4) NNC. All samples underwent standardized electron microscopy preparation beginning with a 30-min fixation in 4 % Glutaraldehyde (GA), followed by three 5-min PBS washes. Subsequent processing included sequential 15-min dehydration in ethanol gradients (30 %, 50 %, 70 %, 90 %, and 100 %), critical point drying, and 10-nm gold sputter coating before field emission SEM examination at 10 kV accelerating voltage.

### 5.9 Mechanical property assessment

To simulate the physiological conditions following *in vivo* implantation, all specimens were preconditioned overnight in PBS at 37 °C before mechanical testing. Evaluations were performed immediately after precondition to reduce environmental influences. The three materials underwent 1000 compression cycles, and a reduction in lumen diameter post-compression was measured. The neural conduits were further secured on a mechanical testing system (BOSS 5100, Massachusetts, USA) to assess the stress variations during a single compression cycle at 50 % lumen deformation. Tensile testing involved evaluating the elongation-conduit deformation relationship, and examining the mechanical behavior of suture knots under tension using 8-0 surgical sutures to replicate an intraoperative suture extraction state.

### 5.10 In vitro cytotoxicity assessment

L-929 cells were maintained and expanded for 2–3 passages in complete MEM (containing 10 % FBS) within a 37 °C, 5 % CO_2_ incubator. Following harvest via trypsinization and centrifugation, a single-cell suspension was prepared in new medium at a density of 1 × 10^5^ cells/mL. A volume of 100 μL of this suspension was added to each well of 96-well plates. The seeded plates were then cultured for 24 h under high-humidity conditions (>90 %) at 37 °C with 5 % CO_2_. Following that, microscopic evaluation of cell morphology was performed to validate proper monolayer establishment and optimal growth conditions.

For sample Preparation, a sufficient amount of the three nerve conduits was placed in a closed inert container under aseptic conditions before adding the extraction medium (MEM medium containing 10 % FBS) at a ratio of 3 cm^2^/mL. Subsequently, the test samples were immersed in the extraction medium and incubated at 37 °C with continuous agitation for 72 h. The physical state of the extraction medium was documented both before and after extraction. All solutions were used within 24 h post-extraction. A fresh extraction medium served as the blank control and was subjected to similar processing conditions as the test samples.

After culturing L-929 cells for 24 h to achieve complete wall attachment, the original medium was replaced with test sample and blank control group extracts. Each group was inoculated into 16 wells, which were incubated at 5 % CO2, Relative Humidity (RH) > 90 %, and 37 °C for 24, 48, 72, and 96 h. At each time point, the culture medium was removed from four replicate wells per group. Then, 50 μl of MTT solution (1 mg/mL) was added to each well, followed by incubation at 37 °C for 2 h in the dark. After carefully removing the MTT solution, 100 μL of isopropanol was added to each well. The plates were gently agitated for 10 min before measuring the absorbance at 570 nm using a TECAN Infinite 200 Pro microplate reader. Cell viability was calculated as a percentage relative to the blank control group using the formula: cell viability% = $\frac{{\text{OD}}_{570,\;test}}{{\text{OD}}_{570,\;blank}}\times 100\%$,where OD_570_, test and OD_570_, blank represent the absorbance values of the test sample and blank control, respectively.

### 5.11 Primary isolation, purification, and expansion of Schwann Cells (SCs)

Postnatal day 3 Sprague-Dawley (SD) rat pups were subjected to 15-min sterilization with 75 % ethanol and humanely sacrificed via cervical dislocation. Using sterile microdissection techniques under a stereomicroscope, bilateral sciatic nerves were isolated along their anatomical course (proximal: sacroiliac articulation; distal: popliteal bifurcation) with final transection at the femoral posterior plane. To remove residual blood components, the isolated sciatic nerve specimens were thoroughly rinsed with DMEM/F-12 medium. The sciatic nerve samples were enzymatically digested at 37 °C for 30 min with gentle agitation in a digestion solution containing trypsin, Col I, and DMEM/F-12 (1:1:9). After centrifugation of the digested samples (400 × g, 3 min, RT), the supernatant was discarded and the resulting pellets were resuspended in 3 mL of SC basal medium. This medium consisted of DMEM/F-12 supplemented with 10 % FBS, 2 mM GlutaMAX-I, and 1 % penicillin-streptomycin. The cell suspensions were then transferred to 35 mm^2^ culture flasks and maintained under standard culture conditions (37 °C, 5 % CO_2_). After 24 h of culture, the medium was replaced with a purification medium containing 10 μM Cytosine Arabinoside (Ara-C) in basal DMEM/F-12 to inhibit fibroblast proliferation, and incubated for an additional 72 h. The medium was then replaced with a complete medium to promote SC proliferation. At 80 %–90 % confluence, the cells were passaged, and 2nd-3rd generation SCs were selected for subsequent experiments [74].

### 5.12 Assessment of SC proliferative capacity

Second-passage SCs were detached using 0.25 % trypsin, centrifuged, and resuspended in complete growth medium. The cells were then seeded at a density of 2000 cells/cm^2^ onto three types of neural conduits and TCP (n = 40). After incubation under standard culture conditions (37 °C, 5 % CO_2_) for 24, 48, 72, and 96 h, 10 wells/group were selected at each time point. Subsequently, the medium was removed and the cells were washed gently with PBS and incubated with 40 % (v/v) CCK-8 solution (HY-K0301, MCE) in complete SC medium for 4 h at 37 °C and 5 % CO_2_. Absorbance was measured at 450 nm using a microplate reader (Infinite 200 Pro, TECAN).

EdU (5-Ethynyl-2′-deoxyuridine) proliferation assay: SCs were seeded onto three types of neural conduits and Tissue Culture Plastic (TCP) controls following the same protocol described above. Following the EdU kit (X0718, Ribobio) instructions, after 24 h of culture in a complete SC medium at 37 °C with 5 % CO_2_, the original culture medium was replaced with complete SC medium containing 50 μM EdU, followed by a 2h incubation under standard culture conditions (37 °C, 5 % CO_2_). The cells were then washed twice with PBS (5 min/wash), followed by sequential fixation with 4 % PFA and permeabilization with 0.5 % Triton X-100 at RT. Subsequently, the cells were treated with Apollo staining reaction cocktail for 30 min at room temperature in the dark. This was followed by 2–3 washes, each lasting 10 min, using 0.5 % Triton X-100. After removing the permeabilization solution, the samples were imaged using either a confocal microscope (LSM 780, Zeiss) or a panoramic confocal slide scanner (Pannoramic, 3DHISTECH).

### 5.13 In vivo assays

Ten millimeter rat sciatic nerve defect models: 60 healthy female SD rats with body weights ranging from 250 to 500 g were used in this study. The rats were randomly assigned to four groups (n = 15): (1) EHNC, (2) non-ECM-modified helical nerve conduit (HNC), (3) NNC, and (4) AUTO (positive control). After body weight measurement, the rats were intraperitoneally injected with pentobarbital sodium (30 mg/kg) to induce anesthesia. Subsequently, the right sciatic nerve was fully exposed, and a 10-mm nerve defect was created. Under surgical microscope guidance (Leica M320×, 10 × magnification), nerve grafts were bridged using tension-free 8-0 nylon sutures (Ethilon™), with precise anastomosis achieved through 1-mm intraneural suture placement at both the proximal and distal stumps. The excised 10-mm sciatic nerve segment was reversed 180° and reimplanted as an autograft (AUTO). After complete hemostasis, the wound was closed in layers using 4-0 sutures followed by povidone-iodine antisepsis. Following surgery, the animals were placed in temperature-controlled recovery chambers until they regained full mobility. They were then housed in standard plastic cages under a controlled ambient temperature of 20–25 °C and a relative humidity of 40–60 percent, with free access to water and a maintained 12-h light/dark cycle.

Anterior tibial trans ankle 25 mm nerve deficit injury model in Beagle dogs: Female beagles aged 12 months and weighing between 7 and 13 kg were used. The animals were weighed and anesthetized via an intramuscular injection of the combination of Zoletil (15 mg/kg) and xylazine hydrochloride (5 mg/kg). After anesthesia induction, the right anterior tibial nerve was completely exposed through anatomical dissection under sterile conditions, with equal separation both above and below the ankle joint, creating a defect of 25–35 mm in length. The anterior tibial nerve was then transected to create a 25 mm anterior tibial nerve defect, into which the EHNC was implanted. The implanted nerve conduits’ flexibility, kink resistance, and stability were systematically evaluated in the beagle model.

Harvesting of Nerve Graft Specimens: At postoperative weeks 3 (short-term) and 12 (long-term), the animals from each group were euthanized via an overdose intraperitoneal injection of pentobarbital sodium. The nerve conduits were then explanted and immersed in 4 % PFA at a 10:1 fixative-to-sample volume ratio, followed by 24-h fixation at 4 °C. Subsequently, the samples were dehydrated in a 30 % sucrose solution and embedded in a composite gel rapidly frozen in liquid nitrogen. The nerve grafts were then cut into 10-μm longitudinal sections.

Histopathological Evaluation of Regenerated Tissues: For Immunofluorescence (IF) staining, the frozen tissue sections were first rinsed with distilled water to remove the embedding compound, blocked with goat serum (SL038, Solarbio, 1:20) at RT for 2 h, incubated with primary antibodies S100-β (ab52642 Abcam)/NF200 (N0142 Abcam) and CD31(ab222783 Abcam)/α-SMA (ab7817 Abcam) at 4 °C for 12 h, and then washed three times with PBS (5 min/wash). Following that, the sections were incubated with secondary antibodies (goat anti-mouse IgG H&L (Alexa Fluor® 488)/goat anti-rabbit IgG H&L (Alexa Fluor® 594)) at RT for 2 h. After washing the staining solution thoroughly with PBS, DAPI solution (10 μg/mL) was added to stain the film for 30 min. The film was subsequently rinsed three times in PBS for 5 min each. After applying an aqueous mounting medium, samples were imaged using a Pannoramic confocal scanner (3DHISTECH) and quantitatively assessed with ImageJ software (v1.52a).

Transmission Electron Microscopy (TEM) analysis: Isolated nerve tissue blocks of the middle segment of the regenerating nerve were first fixed in a 4 °C electron microscopy fixative for 4 h and washed with boric acid buffer three times (10 min/wash). After rinsing, the samples were fixed with 1 % osmium tetroxide in 0.1 M buffer for 2 h and dehydrated with a graded ethanol series (50 %–100 %). Following that, the samples were transferred to 100 % acetone, permeabilized overnight, and resin-embedded for 48 h. The samples were then sliced into 60-80 nm-thick sections using an ultramicrotome. After double staining with uranyl acetate and lead citrate, the morphology of axons and myelin sheaths was photographed with a transmission electron microscope. Using ImageJ software, six Fields of View (FOV) were randomly selected in each group of samples to determine myelin thickness and nerve fiber diameter, respectively.

Gait analysis: Footprints of each group of experimental animals (n = 5) were imaged using an animal gait analyzer (Catwalk, Noldus) at 3 and 12 weeks post-surgery. Based on the animal's walking tracks, three footprint parameters were measured separately for the injured (E) and normal (N) sides: Toe width (TS), intermediate toe width (ITS), and footprint length (PL). The Sciatic Function Index (SFI) was calculated using the CatWalk XT 10.6 (Noldus) software.

Electrophysiology: At 12 weeks post-surgery, body weights of rats in each group were measured before anesthetizing the animals via intraperitoneal injection of sodium pentobarbital (30 mg/kg). After anesthesia induction, the left intact sciatic nerve and the right nerve conduit were sequentially dissected and exposed. The animals were then subjected to neurophysiological assessments using a synergistic electromyography recording system (Oxford). The stimulating electrode was placed at the graft's proximal end, while the recording electrode was fixed parallel to the surface of the gastrocnemius muscle belly. The stimulation parameters were set to a constant current of 3 mA, with the single stimulation-evoked Compound Muscle Action Potential (CMAP) recorded. The left intact sciatic nerve was tested in the same manner and served as an internal control. After data collection, CMAP latency and peak amplitude ratios (surgical side/control side) were analyzed and compared across groups.

Pathologic evaluation of target muscle tissue: The rats’ bilateral gastrocnemius muscles were excised under anesthesia, weighed individually, and subsequently immersed in 4 % PFA at a volume 10 times that of the tissue for 48 h of fixation. Subsequently, the muscles were routinely dehydrated and embedded in paraffin before preparing 4-μm transverse sections using a paraffin microtome. For pathological staining, tissue sections were processed with a Masson staining kit (G1006, Servicebio). Following deparaffinization through two cycles of xylene (5–10 min per cycle), tissue sections were progressively rehydrated using a descending ethanol gradient (100 %, 95 %, 85 %, and 75 %; 3 min per concentration) and subsequently washed in deionized water for 2 min. Hematoxylin staining was carried out for 5 min, after which unbound dye was removed by rinsing with distilled water. The sections were briefly treated with acidic differentiation solution (30 s) and then immersed in tap water for 10 min to develop the blue coloration. Subsequently, tissue sections were treated with Ponceau S-acid fuchsin solution for 10 min, subjected to a brief distilled water rinse, and differentiated in phosphomolybdic acid solution for 2 min. Subsequently, the samples were counterstained with brilliant green for 1 min, washed with distilled water, and immersed in acidic differentiation solution for 1 min for further differentiation. Finally, the sections underwent rapid dehydration through a graded ethanol series (75 %, 85 %, 95 %, and 100 %; 10 s per concentration) followed by xylene clearing and neutral balsam mounting prior to whole-slide imaging using a panoramic scanner.

### 5.14 Image analysis and statistical analysis

All experiments were performed with at least three independent replicates. Image analysis was conducted using Image Pro Plus 6.0 software. Statistical analyses were performed with GraphPad Prism 7.0, and data are presented as the mean ± standard deviation (X ± s). For multiple group comparisons, one-way ANOVA was applied, followed by Student's t-test for comparisons between two specific groups. A p-value of ≤0.05 was considered statistically significant.

## CRediT authorship contribution statement

**Yixiao Tan:** Writing – review & editing, Writing – original draft, Software, Methodology, Formal analysis, Data curation. **Jiafeng Yi:** Visualization, Methodology, Formal analysis, Data curation. **Pengtao Shi:** Software, Methodology, Formal analysis, Data curation. **Qingda Wei:** Visualization, Software, Methodology, Formal analysis, Data curation. **Yuhui Cui:** Visualization, Data curation. **Yanjun Guan:** Formal analysis, Data curation. **Haofeng Cheng:** Formal analysis, Data curation. **Tianqi Su:** Formal analysis, Data curation. **Qianru Yao:** Formal analysis, Data curation. **Haolin Liu:** Software, Methodology. **Ruichao He:** Software, Formal analysis. **Junli Wang:** Visualization, Software. **Yiben Ouyang:** Data curation. **Xiaoyang Fu:** Visualization. **Jinjuan Zhao:** Data curation. **Yu Wang:** Writing – review & editing, Writing – original draft, Validation, Supervision, Resources, Investigation, Funding acquisition. **Qi Quan:** Writing – review & editing, Writing – original draft, Supervision, Resources, Project administration, Investigation, Funding acquisition. **Wei Chai:** Writing – review & editing, Writing – original draft, Supervision, Resources, Project administration, Investigation, Funding acquisition.

## Ethics statement

This study obtained written approval from the Ethics Committee of the Chinese PLA General Hospital, Beijing, China.The animal experiment ethics approval number is 2023-x4-19.

## Funding

This work was supported by the grants from the Natural Science Foundation of Beijing Municipality (L244029, L244058, L242137), 10.13039/501100001809National Natural Science Foundation of China (82425036,32301111).

## Declaration of competing interest

The authors declare that they have no known competing financial interests or personal relationships that could have appeared to influence the work reported in this paper.

## Data Availability

No data was used for the research described in the article.

## Appendix B Supplementary data

Video S1A Video S1A

Video S1B Video S1B

Video S1C Video S1C

Video S2A Video S2A

Video S2B Video S2B

Video S2C Video S2C

Video S3A Video S3A

Video S3B Video S3B

Video S3C Video S3C
